# Followership among jordanian nurses: A cross-sectional online study

**DOI:** 10.1371/journal.pone.0339008

**Published:** 2025-12-23

**Authors:** Wafaa T. ALQadrie, Ali M. Saleh, Sami AL-Rawashdeh, Ola N. Alfuqaha, Suheir I. Abdallah

**Affiliations:** 1 Community Health Nursing Department, School of Nursing, The University of Jordan, Amman, Jordan; 2 Community and Mental Health Nursing Department, Faculty of Nursing, The Hashemite University, Zarqa, Jordan; Eötvös Loránd University, HUNGARY

## Abstract

**Background:**

In recent years, the concept of followership has gained significant attention, challenging the traditional leadership-centric view of organizational management positions. While effective followership is vital to the success of any organization, it has often been overlooked, especially in healthcare, where teamwork is crucial.

**Aim:**

The current study aimed to identify the followership styles of nurses in Jordan and examine how these styles relate to nurses’ demographic and work-related characteristics.

**Method:**

A descriptive cross-sectional design was employed. Using a multistage sampling approach, 351 registered nurses were recruited from governmental, private, and teaching hospitals across northern, central, and southern Jordan. Data were collected through an online survey using the validated Kelley Followership Questionnaire–Revised (KFQ-R). Both descriptive and inferential statistical analyses were conducted.

**Results:**

Findings revealed that the predominant followership style was exemplary (68.7%), followed by the pragmatist (31.3%). Chi square test revealed statistically significant difference between followership style and marital status (χ² (1) = 6.193, p = .013), working area (χ² (2) = 7.405, p = .025), nursing care delivery system (χ² (3) = 13.926, p = .003), and Decision-making style (χ² (3) = 17.173, p = .001). The binary logistic regression was significant, χ² (26) = 67.77, p < .001, identifying participatory decision-making style, working area, and nursing care delivery system as significant predictors of exemplary followership.

Conclusion

The study found that nurses working in participatory decision-making environments, team-based models of care, and high-intensity units such as emergency departments were more likely to exhibit the characteristics of exemplary followers. Nurse leaders and managers should foster work environments that promote autonomy, support shared decision-making, and encourage active team engagement. These findings emphasize the critical role of effective followership in advancing collaborative practice, optimizing clinical decision-making, and strengthening workforce performance, underscoring its integration into nursing education, leadership development program, and organizational policy.

## Introduction

Followership refers to the constellation of behaviors, cognitions, and interpersonal competencies that enable individuals to align with shared objectives and contribute meaningfully to collective performance [1,2]. In clinical care, where interdependent teams work under uncertainty and time constraints, followership is not peripheral; it is essential to safe and reliable practice [3,4]. Nurses coordinate patient monitoring, implement medical orders, escalate emerging concerns, and identify latent safety threats through inquiry and advocacy [5]. These activities extend beyond procedural compliance and require independent clinical judgment, sustained situational awareness, and purposeful, constructive engagement across professional boundaries. Despite this centrality, followership has historically received less attention than leadership within health‑care scholarship and organizational development [4,6,7]. Leader‑centric paradigms can obscure the reality that high‑functioning units rely on fluid, reciprocal role shifts: the same clinicians lead in one moment and follow in the next, depending on expertise, proximity to the problem, and the situational stakes [3,7]. Positioning followership as an active, competency-based role rebalances the discourse and establishes it as a focus for curricular development and organizational design [6,7].

Misconceptions have impeded the uptake of the concept. The term “follower” has at times been equated with passivity, deference, or a lack of agency, which can discourage self‑identification and undermine developmental efforts [8–10]. Kelley’s widely cited reframing counters this pejorative reading by emphasizing two orthogonal dimensions independent critical thinking and active engagement that together describe how individuals contribute in follower roles. Combined, these dimensions yield five styles: exemplary (high thinking, high engagement), alienated (high thinking, low engagement), conformist (low thinking, high engagement), passive (low‑low), and pragmatist (moderate on both) [8,9]. Crucially, the model conceptualizes followers as capable, discerning partners who can shape leaders’ behavior through constructive challenge, upward influence, and reliable execution. In nursing, this perspective reflects everyday practice: nurses appraise clinical directives in light of evolving patient presentations, interrogate ambiguities in orders, and advocate during handovers or periods of rapid clinical change [1,10]. Emerging empirical evidence in nursing further indicates that exemplary and pragmatist followership styles are most frequently observed, although their distribution varies across settings and specialties [11,12].

Studies in early‑career cohorts have reported a predominance of exemplary styles [11,13], while critical‑care and acute‑care environments often exhibit a mix of exemplary and pragmatist patterns with comparatively few passive or alienated classifications [12]. These findings, while promising, cannot be assumed to generalize across systems. Health‑care structures, professional hierarchies, and cultural norms shape how discretion, voice, and engagement are enacted [1]. In settings where decision authority is centralized or team communication is highly formal, certain styles may be more adaptive or at least more visible than others. This dynamic is particularly salient in Jordan, where nursing practice operates within pronounced hierarchical structures that influence when and how nurses speak up, question decisions, or participate in shared decision-making [14–16]. Moreover, the scarcity of empirical followership research in Middle Eastern healthcare contexts underscores the need for context-specific inquiry, positioning the Jordanian nursing workforce as a compelling setting for examining how followership styles manifest within such organizational and cultural frameworks.

Nurses constitute the largest segment of the healthcare workforce in Jordan and bear substantial responsibility for direct patient care across government, private, military, and university-affiliated hospitals [14]. Within these settings, hierarchical authority gradients continue to shape communication patterns and escalation pathways [15,16]. At the same time, increasing patient acuity, multimorbidity, and pressures on hospital throughput place a premium on vigilant surveillance, interprofessional coordination, and timely advocacy—behaviours strongly aligned with active and critically engaged followership. Understanding how Jordanian nurses distribute across Kelley’s followership styles can therefore inform workforce development, unit-level quality improvement, and pre-licensure education, while also revealing whether internationally observed patterns hold in this context and where targeted interventions may strengthen team functioning [8].Beyond descriptive value, a rigorous examination of followership in Jordan addresses a practical challenge for managers and educators: most organizational investments focus on developing formal leaders, while the majority of staff enact follower roles for much of their workday [7]. If units are staffed by nurses who are engaged but hesitant to question, or by nurses who think critically but disengage behaviorally, the resulting mismatch can blunt the effects of leadership initiatives and dampen safety culture. Conversely, cultivating exemplary followership where independent thinking and active contribution coincide may amplify the impact of leadership and provide redundancy in the detection and mitigation of risk [2,4,5,9,11,12]. A national study using a validated instrument can surface these patterns and offer a baseline against which future interventions are evaluated.

The present study responds to this need. It aims to characterize the distribution of followership styles among registered nurses working in hospitals across Jordan and to examine how style classifications vary by demographic and work‑related characteristics. Kelley’s model provides the conceptual frame, and the revised Kelley Followership Questionnaire (KFQ‑R) serves as the measurement instrument [8,9,17]. Because language and cultural nuance matter in self‑report tools, an Arabic version of the KFQ‑R was translated and content‑validated prior to data collection to ensure conceptual equivalence and clarity. The overarching goal is to generate practical, context‑sensitive evidence that can guide policy development, education, and team design initiatives in Jordanian hospitals. To achieve this purpose, the study addressed the following research questions:

1. What are the predominant followership styles among nurses working in Jordanian hospitals?
2. Are there significant differences in followership styles according to nurses’ demographic and workplace-related characteristics?
3. What factors predict followership styles among nurses in Jordan?

## Materials and methods

### Study design and setting

We conducted a descriptive cross‑sectional survey across multiple regions and hospital sectors in Jordan. A multistage cluster sampling strategy was used to achieve broad geographic and institutional representation. First, the country was stratified into three regions (north, middle, south). Within each region [14], hospitals were stratified by ownership type government, private, and university‑affiliated and a proportional number of hospitals were randomly selected from each stratum. To ensure a minimum staffing base for recruitment, eligibility at the facility level requires at least 60 inpatient beds. Fifteen hospitals ultimately participated, reflecting the distribution of sizable institutions across regions and sectors. This design balances feasibility with representativeness and is well suited to describing national patterns in work attitudes and behaviors [18].

### Participants and eligibility

Eligible participants were registered nurses who held at least a baccalaureate degree, had accrued six months or more of clinical experience in their current hospital, and provided informed consent electronically. Nurses in primarily administrative roles or those assigned exclusively to outpatient clinics were excluded to keep the focus on inpatient unit dynamics where interprofessional coordination and escalation behaviors are most salient. Proportionate allocation was used to set recruitment targets for each hospital. Within each hospital, nursing units were randomly selected across major clinical areas—including adult medical–surgical wards, critical care, emergency, obstetrics and gynecology, pediatrics, and operating rooms—and all nurses working in those units were invited to participate. Given variable shift patterns, workload demands, and limited direct access to staff, this approach offered a feasible recruitment method while capturing diversity in workload, patient acuity, and team composition that could plausibly influence followership.

### Sample size justification

For the binary logistic regression analysis, sample size adequacy was evaluated using the events-per-variable (EPV) rule of thumb, in line with recent methodological recommendations that suggest an EPV ≥ 20 to reduce bias in regression coefficients and improve predictive accuracy [19]. Based on the inclusion of 13 predictors in the model and the conservative criterion of 25 events per variable, a minimum sample size of approximately 325 participants was required. The achieved sample of 351 nurses exceeded this threshold, indicating that the logistic regression analysis was adequately powered and that the coefficient estimates are likely to be stable.

### Measures

Data were collected using a two‑part electronic questionnaire. The first part captured demographic and work‑related characteristics: gender, age, marital status, highest educational attainment, monthly income bracket, total years in nursing, tenure on the current unit, clinical area of practice, geographic region and hospital type, shift model, the perceived decision‑making style on the unit, and prior knowledge of one’s followership style. These variables were selected based on theoretical relevance to engagement and voice behaviors and to enable comparisons with international studies.

The second part operationalized followership using the KFQ‑R [17]. The instrument comprises 25 items rated on a 7‑point scale (0–6) and yields two subscales aligned with Kelley’s model independent critical thinking (13 items; total possible score 0–78) and active engagement (12 items; total possible score 0–72). The survey instrument is provided as S1 File. Followership styles were classified in accordance with Kelley’s two-axis model of independent critical thinking and active engagement [9], using score ranges adapted for the KFQ-Revised [17]. Participants scoring ≥53 on independent critical thinking and ≥49 on active engagement were classified as Exemplary. Scores ≥53 on critical thinking but <24 on engagement were categorized as Alienated. Participants scoring <26 on critical thinking and ≥49 on engagement were classified as Conformist, whereas those scoring <26 on both dimensions were classified as Passive. Mid-range values (approximately 26–52 for critical thinking and 24–48 for engagement) corresponded to the central Pragmatist region, consistent with Kelley’s conceptualization of average followers [17]. Prior psychometric work reports strong internal consistency for the overall instrument and excellent reliability for both subscales [8,9,11,17]. In the present study, the Arabic translation demonstrated good internal consistency for the total scale and satisfactory coefficients for the subscales, supporting its use in the Jordanian context.

#### Translation and content validation.

Translation followed established guidance for cross‑cultural adaptation of self‑report measures. A bilingual subject‑matter expert produced a forward translation from English to Arabic [20]. An independent bilingual translator blinded to the original completed a back‑translation to English. A panel of six reviewers, including four PhD‑prepared nurse leaders and two senior clinical academics, adjudicated discrepancies and refined wording to optimize conceptual equivalence and readability for hospital nurses in Jordan. The panel evaluated each item for relevance and clarity, and item‑level content validity indices (I‑CVI) ranged from 0.83 to 1.00. The scale‑level CVI/Average was 0.94, indicating high overall content validity [21]. Minor wording adjustments were implemented to reduce ambiguity while preserving the constructs represented by the original items.

To evaluate the clarity, reliability, and cultural relevance of the Arabic version, a pilot study was carried out with 35 registered nurses (around 10% of the total sample) who met the inclusion criteria. The results showed strong internal consistency for the KFQ-R, with a Cronbach’s alpha coefficient of 0.89, indicating that the items were reliably measuring the intended construct. To maintain the integrity of the main study, data from the pilot participants were not included in the final analysis.

#### Procedures and data collection.

A secure online survey was hosted on Google Forms. The platform was configured to enforce single submission per participant and included filtering questions to confirm eligibility prior to item display. Recruitment messages were disseminated through professional networks and WhatsApp groups commonly used by nurses in Jordan, with a reminder message one week after initial distribution. The survey contained 39 items (25 KFQ‑R items and 14 demographic/work items) and required approximately 10 minutes to complete. The invitation letter included the primary researcher’s contact information. The form closed after 351 complete responses were obtained. Required fields and range checks minimized missing data and improved data quality at the point of entry. Data were collected between April 1 and June 1, 2025.

#### Ethical considerations.

This study was reviewed and approved by the Scientific Research Ethics Committee of the Ministry of Health, Hashemite Kingdom of Jordan (Approval No. MOH/REC/2024/114). Participation was voluntary. The consent statement, presented on the first survey page, described the study purpose, procedures, risks, and benefits; participants affirmed consent electronically before proceeding. No identifying information was collected beyond optional contact details for questions, and all data were stored on password‑protected devices accessible only to the research team. Participants were informed that they could exit the survey at any time prior to submission without penalty. Permission to use the KFQ-R was obtained directly from the primary author before data collection.

### Data management and analysis

Data were exported to IBM SPSS Statistics (version 26) for cleaning and analysis. Quality checks included verification of valid score ranges for the KFQ‑R items, confirmation of single submissions, and review for implausible values. Descriptive statistics summarized participant characteristics and the distribution of independent critical thinking and active engagement scores. Style categories were assigned using subscale cut‑points consistent with the KFQ‑R guidance, and frequency distributions were calculated for the five categories [17]. Associations between style categories and demographic or work variables were examined with chi‑square tests at a significance threshold of 0.05. Binary logistic regression was performed to examine the association between demographic, professional, and organizational factors and the likelihood of nurses exhibiting an exemplary followership style. Internal consistency reliability for the translated instrument was assessed using Cronbach’s alpha for the total scale and each subscale.

This study was conducted in accordance with relevant reporting guidelines. The STROBE checklist is available as S2 File.

## Results

### Socio-demographic characteristics of participants

The study participants had a mean age of 32.4 years (SD = 5.4), with ages ranging from 22 to 41 years. The participants had a mean number of years of experience in the nursing profession of 9.2 years (SD = 5.2), while the mean years of experience in their current unit or ward was 5.8 years (SD = 4.3). The majority of participants were middle-aged employees with significant experience in their roles. The study participants’ monthly income ranged from JOD 250–1000 (USD 352–1400), with a mean of 573 JOD (SD = 158), reflecting the economic diversity within the nursing workforce. In regard to the gender, the study sample included 143 male nurses (40.7%) and 208 female nurses (59.3%), indicating a relatively balanced gender representation of Jordanian workforce compared to the typically female-dominated nursing workforce in many regions. The majority of respondents were married (63.2%), while 33.9% were single and 2.8% were divorced or separated. Regarding educational qualifications, 88.6% held a bachelor’s degree, 10% held a master’s degree, and a small proportion, 1.4%, had a PhD.

Participants were distributed across various working areas. The largest proportion worked in general wards (medical, surgical, maternal, etc., 44.7%), followed by intensive care units (28.8%), and emergency departments (26.5%). This reflects a wide range of clinical exposure among participants. The majority (79.8%) reported working rotating shifts, while 20.2% worked fixed shifts.

Regarding hospital type, 46.2% were employed in governmental hospitals, 27.9% in teaching-affiliated hospitals, and 25.9% in private hospitals, indicating a broad representation from different healthcare sectors. In terms of model of nursing care, the most common model of nursing care was Total patient care (38.5%), followed closely by team nursing (37.9%), while functional care (18.8%) and other/unclear models (4.8%) were less prevalent. This diversity may influence teamwork dynamics and followership behavior. Regarding decision-making styles in their units, 37.3% reported a participative (bilateral) approach, 36.2% reported a mixed style, and 19.1% perceived an authoritative-unilateral approach, while 7.4% were uncertain. The study sample was distributed almost across all three districts. The majority of participants (n = 186, 53.0%) were from the Middle region of Jordan. This was followed by 105 participants (29.9%) from the Northern region. The Southern region accounted for the smallest proportion, with 60 nurses (17.1%). Table 1 shows details about the study participants’ demographic characteristics.

**Table 1:** Description of participants’ characteristics (N = 351).

Variable	M (SD) or n (%)	Range (Min–Max)
Age (years)	32.4 (5.4)	22–41
Years of experience in nursing profession	9.2 (5.2)	1–20
Years of experience in current unit/ward	5.8 (4.3)	1–19
Monthly income (JOD)	573 (158)	250–1000
Gender		
Male	143 (40.7)	—
Female	208 (59.3)	—
Marital status		
Married	222 (63.2)	—
Single	119 (33.9)	—
Divorced/Separated	10 (2.8)	—
Education level		
Bachelor	311 (88.6)	—
Master	35 (10.0)	—
PhD	5 (1.4)	—
Working area		
Wards (medical, surgical, maternal, etc.)	157 (44.7)	—
Intensive Care Units (ICUs)	101 (28.8)	—
Emergency Department	93 (26.5)	—
Shift type		
Rotating shift (A, B, C, Day, Night)	280 (79.8)	—
Fixed shift (A, Day, C, Night)	71 (20.2)	—
Type of hospital		
Governmental	162 (46.2)	—
Teaching affiliated	98 (27.9)	—
Private	91 (25.9)	—
Model of nursing care provision		
Total nursing care	135 (38.5)	—
Team nursing care	133 (37.9)	—
Functional nursing care	66 (18.8)	—
Unclear	17 (4.8)	—
Decision-making style		
Authoritative–unilateral	67 (19.1)	—
Participating–bilateral	131 (37.3)	—
Mixed	127 (36.2)	—
Unclear	26 (7.4)	—
District geographical region		
North	105 (29.9)	—
Middle	186 (53.0)	—
South	60 (17.1)	—
If you know your followership style		
Yes	139 (39.6)	—
No	212 (60.4)	—

Note: Values are presented as mean (SD) or frequency (percentage). JOD = Jordanian Dinar.

### Issssnstrument reliability and validity

A sufficiently large sample of 351 participants allowed for examination of the psychometric properties of the scale and its subscales. Cronbach’s alpha coefficients were computed for the total scale and its subscales. The Arabic version of the Kelly Followership Questionnaire–Revised (KF-R) showed excellent internal consistency (α = .91). The KF-R comprises two dimensions: independent critical thinking (α = .84) and active engagement (α = .87).

### The predominant followership styles among nurses in Jordanian hospitals

#### Followership styles.

The participants were asked if they were aware of their own followership style, about two-thirds of subjects (60.4%) indicated that they were not aware of their own followership style. The participants’ followership style was assessed based on Kelley’s followership model, which categorizes followers along two dimensions: independent critical thinking and active engagement [8,9]. Based on these dimensions, five distinct followership styles are typically identified: exemplary, pragmatic, alienated, conformist, and passive. The current study measured total scores on independent critical thinking and active engagement to classify respondents accordingly.

As presented in Table 2, of the 351 participants, 68.7%, and 31.3%, had exemplary, and pragmatist followership styles, respectively. The conformist, alienated, and passive followership styles were not found. In addition, the mean scores further support these classifications as Total Independent Critical Thinking scores mean was 55.5 (SD = 11.3), and Total Active Engagement scores mean was 54.9 (SD = 10.8). These high mean scores across both dimensions indicate that the majority of participants are strongly aligned with the characteristics of exemplary followership.

**Table 2:** Followership styles among study participants (N = 351).

Variable	Mean (±SD)	Range (Min–Max)	Frequency (%)
Total independent critical thinking	55.5 (±11.3)	52 (26–78)	—
Total active engagement	54.9 (±10.8)	47 (25–72)	—
Followership style			
Exemplary			241 (68.7)
Pragmatist			110 (31.3)
Alienated			0
Conformist			0
Passive			0

Note: Values are presented as mean (±SD) or frequency (percentage).

### Differences in followership styles across demographic and work-related characteristics

To answer the second research question in the current study, chi-square tests of independence were conducted. This non-parametric test is appropriate for analyzing relationships between categorical variables, as it determines whether the distribution of one variable differs across the categories of another [22]. Chi-square tests allowed for the assessment of difference between categorical variables, specifically the classification of followership styles (exemplary vs pragmatic) as dependent variable and various demographic factors were categorical variables. Assumptions of Chi-square were tested: the independent observations and a minimum of 80% of the cells with an expected frequency of not less than 5. The analysis included 351 valid responses, with no missing data. A summary of these differences is provided in Table 3.

**Table 3:** Differences in followership styles among nurses in Jordan based on demographics and workplace-related characteristics (N = 351).

Variable	Exemplary n (%)	Pragmatist n (%)	Test value	df	p-value
Age					
< 25 years (n = 37)	29 (78.4)	8 (21.6)	χ² = 2.041	2	0.357
25–34 years (n = 173)	119 (68.8)	54 (31.2)			
≥ 35 years (n = 141)	93 (66.0)	48 (34.0)			
Years of experience (nursing)					
1–2 (n = 53)	38 (71.7)	15 (28.3)	χ² = 5.78	3	0.123
3–4 (n = 31)	19 (61.3)	12 (38.7)			
5–9 (n = 86)	67 (77.9)	19 (22.1)			
≥ 10 (n = 181)	117 (64.6)	64 (35.4)			
Years of experience in current unit/ward					
1–2 (n = 98)	66 (67.3)	32 (32.7)	χ² = 7.56	3	0.056
3–4 (n = 73)	51 (69.9)	22 (30.1)			
5–9 (n = 101)	78 (77.2)	23 (22.8)			
≥ 10 (n = 79)	46 (58.2)	33 (41.8)			
Monthly income (JOD)					
≤ 500	116 (73.0)	43 (27.0)	χ² = 3.25	2	0.203
501–700	93 (66.9)	46 (33.1)			
> 700	32 (60.4)	21 (39.6)			
Gender					
Male (n = 143)	101 (70.6)	42 (29.4)	χ² = 0.435	1	0.510
Female (n = 208)	140 (67.3)	68 (32.7)			
Marital status					
Married (n = 222)	142 (63.9)	80 (36.0)	χ² = 6.19	1	0.013
Single/Separated (n = 129)	99 (76.7)	30 (23.3)			
Education level					
Bachelor (n = 311)	215 (69.1)	96 (30.9)	χ² = 0.281	1	0.596
Master/PhD (n = 40)	26 (65.0)	14 (35.0)			
Working area					
ICU (n = 101)	61 (60.4)	40 (39.6)	χ² = 7.40	2	0.025
ER (n = 93)	73 (78.5)	20 (21.5)			
Wards (n = 157)	107 (68.1)	50 (31.9)			
Shift type					
Fixed (n = 71)	43 (60.6)	28 (39.4)	χ² = 2.71	1	0.115
Rotating (n = 280)	198 (70.7)	82 (29.3)			
Type of hospital					
Private (n = 91)	68 (74.7)	23 (25.3)	χ² = 2.34	2	0.310
Teaching-affiliated (n = 98)	67 (68.7)	31 (31.6)			
Governmental (n = 162)	106 (65.4)	56 (34.6)			
Model of nursing care provision					
Primary (n = 135)	86 (63.7)	49 (36.3)	χ² = 13.92	3	0.003
Team (n = 133)	100 (75.2)	33 (24.8)			
Functional (n = 66)	49 (74.2)	17 (25.8)			
Unclear (n = 17)	6 (35.3)	11 (64.7)			
Decision-making style					
Authoritative–unilateral (n = 67)	34 (50.7)	33 (49.3)	χ² = 17.17	3	0.001
Participatory–bilateral (n = 131)	98 (74.8)	33 (25.2)			
Mixed (n = 127)	95 (74.8)	32 (25.2)			
Unclear (n = 26)	14 (53.8)	12 (46.2)			
District geographical region					
North (n = 105)	80 (76.2)	25 (23.8)	χ² = 5.37	2	0.068
Middle (n = 186)	118 (63.4)	68 (36.6)			
South (n = 60)	43 (71.7)	17 (28.3)			
If you know your followership style					
Yes (n = 139)	92 (66.2)	47 (33.8)	χ² = 0.655	1	0.418
No (n = 212)	149 (70.3)	63 (29.7)			

Note: Data are presented as frequencies (percentages). χ² = Pearson Chi-square test. JOD = Jordanian Dinar

#### Marital status and followership style.

The results revealed a statistically significant association between marital status and followership style, χ²(1) = 6.193, p = .013 (Table 3). Crosstabulation indicated that a higher proportion of single or separated nurses (76.7%) were classified as Exemplary High followers compared to their married counterparts (64.0%). Conversely, married nurses were more likely to be Pragmatist Moderate followers (36.0%) than single/separated nurses (23.3%).

#### Type of working area and followership style.

A statistically significant difference was also observed between unit type and followership style, χ² (2) = 7.405, p = .025. The results revealed that nurses working in Emergency Rooms (ER) were more likely to exhibit Exemplary followership style 78.5% compared to those working in general ward 68.2% and intensive care unit 60.4%. In contrast, nurses in intensive care units were more likely to exhibit Pragmatist style 39.6%.

#### Model of nursing care provision and followership style.

The data also showed a significant statistical difference between model of nursing care provision and followership style, χ² (3) = 13.926, p = .003. Specifically, Nurses who work under team nursing (75.19%) and functional nursing (74.24%) are more likely to be Exemplary followers [20]. Conversely, those reported “Unclear models of care” were more likely to be Pragmatist followers 64.71%.

#### Decision-making style and followership style.

A significant statistical association was found between decision-making style and followership styles, χ²(3) = 17.173, p = .001. Specifically, nurses who reported that they are working in authoritative-unilateral environments where managers control decisions without staff input were more likely to be Pragmatist followers (49.25%) than Nurses who are working in participatory and mixed decision-making environment (25.19%) [23]. In contrast, participating-bilateral and mixed decision-making environments were more associated with Exemplary followership style (74.80%).

Other demographic and work-related characteristics were not significantly associated with followership style (p > .05; see Table 3).

### Predictors of followership styles among nurses in Jordan

Binary logistic regression was performed to identify independent predictors of exemplary followership (coded = 1) versus a pragmatist style (coded = 0). The overall model was statistically significant, χ²(26) = 67.77, p < .001, indicating that the predictors reliably distinguished between the two followership styles. Model fit indices demonstrated acceptable performance (Cox & Snell R² = .176; Nagelkerke R² = .247). The Hosmer–Lemeshow test was non-significant (χ²(8) = 6.44, p = .598), confirming good model fit. The model correctly classified 73.2% of cases, with strong accuracy for identifying exemplary followers (90.0%) and moderate accuracy for pragmatist followers (36.4%).

In the adjusted logistic regression model, three variables significantly predicted exemplary followership: working area, nursing care delivery system, and decision-making style.

### Working area

Nurses in emergency departments had higher odds of exemplary followership than those in ICUs (OR = 2.18, 95% CI: 1.05–4.51, p = 0.036).

### Nursing care delivery system

Team nursing (OR = 10.23, 95% CI: 2.47–42.32, p = 0.001) and functional models (OR = 12.71, 95% CI: 2.79–57.86, p = 0.001) were strongly associated with exemplary followership compared with unclear models.

### Decision-making style

Participatory–bilateral decision-making significantly increased the likelihood of exemplary followership (OR = 2.34, 95% CI: 1.08–5.07, p = 0.032).

No other demographic or work variables were statistically significant predictors (p > 0.05; Table 4).

**Table 4:** Logistic regression models of Followership styles (n = 351).

Background variables	B	S.E.	Wald	df	Sig.	Exp(B)	CI for Exp (B) 95% C.I.	
							Lower	Upper
Age								
< 25 years (n = 37)	−0.146	0.777	0.035	1	0.851	0.864	0.189	3.958
25–34 years (n = 173)	−0.504	0.382	1.738	1	0.187	0.604	0.286	1.278
≥ 35 years (n = 141)								
Years of experience (nursing)								
1–2 (n = 53)	−0.093	0.745	0.015	1	0.901	0.911	0.212	3.926
3–4 (n = 31)	−0.277	0.612	0.205	1	0.651	0.758	0.229	2.515
5–9 (n = 86)	0.528	0.435	1.476	1	0.224	1.696	0.723	3.977
≥ 10 (n = 181)								
Years of experience in current ppunit/ward								
1–2 (n = 98)	0.085	0.478	0.031	1	0.859	1.089	0.426	2.780
3–4 (n = 73)	0.739	0.446	2.736	1	0.098	2.093	0.872	5.021
5–9 (n = 101)	0.792	0.417	3.611	1	0.057	2.207	0.975	4.993
≥ 10 (n = 79)								
Monthly income (JOD)								
≤ 500	0.155	0.463	0.112	1	0.738	1.167	0.471	2.892
501–700	−0.014	0.399	0.001	1	0.972	0.986	0.451	2.155
> 700								
Gender								
Female (n = 208)	0.218	0.298	0.537	1	0.464	1.244	0.694	2.229
Male (n = 143)	Ref.							
Marital status								
Single/Separated (n = 129)	−0.660	0.342	3.714	1	0.054	0.517	0.264	1.011
Married (n = 222)	Ref.							
Education level								
Bachelor (n = 311)	−0.176	0.423	0.173	1	0.677	0.839	0.366	1.922
Master/PhD (n = 40)	Ref.							
Working area								
ICU (n = 101)	−0.093	0.314	0.088	1	0.767	0.911	0.493	1.685
ER (n = 93)	0.779	0.371	4.419	1	0.036	2.180	1.054	4.507
Wards (n = 157)	Ref.							
Shift type								
Rotating (n = 280)	−0.513	0.348	2.171	1	0.141	0.599	0.303	1.184
Fixed (n = 71)	Ref.							
Type of hospital								
Private (n = 91)	−1.006	0.624	2.601	1	0.107	0.366	0.108	1.242
Teaching-affiliated (n = 98)	−0.006	0.603	0.000	1	0.993	0.994	0.305	3.244
Governmental (n = 162)	Ref							
Model of nursing care provision								
Primary (n = 135)	1.514	0.729	4.308	1	0. 382	1.544	1.088	18.975
Team (n = 133)	2.326	0.724	10.306	1	0.001	10.233	2.474	42.328
Functional (n = 66)	2.543	0.773	10.817	1	0.001	12.715	2.794	57.863
Unclear (n = 17)	Ref.							
Decision-making style								
Authoritative–unilateral (n = 67)	0.188	0.368	0.261	1	0.609	1.207	0.587	2.483
Participatory–bilateral (n = 131)	0.848	0.396	4.601	1	0.032	2.336	1.076	5.072
Mixed (n = 127)	0.068	0.597	0.013	1	0.909	1.071	0.332	3.451
Unclear (n = 26)	Ref.							
District geographical region								
North (n = 105)	0.060	0.499	0.014	1	0.905	1.062	0.399	2.825
Middle (n = 186)	−0.796	0.435	3.360	1	0.067	0.451	0.192	1.057
South (n = 60)	Ref.							

Note; Sig., Significant P < 0.05, Ref., Reference category; CI., Confidence Interval

## Discussion

This study provides new insights into the nature of followership among nurses in Jordan, highlighting the perceptions, patterns, and contextual factors that shape how nurses engage within healthcare teams. As the first investigation to examine followership within Jordan’s healthcare system, particularly in the nursing field, this research fills an important gap in understanding how nurses perceive and enact their roles as followers in a hierarchically structured environment.

The findings of this study reveal that the majority of registered nurses in Jordanian hospitals demonstrated an exemplary followership style (68.7%), whereas the remaining 31.3% were pragmatist followers. Notably, none of the respondents fell into the conformist, passive, or alienated categories. This pattern may reflect the organizational context of Jordanian hospitals, where hierarchical structures and strong norms of compliance may limit the expression of these followership styles. Additionally, the voluntary and self-reported nature of the survey may have introduced social desirability bias, particularly in hierarchical clinical environments, potentially shifting responses toward the exemplary range.

The prevalence of high number of exemplary followership might be a result of persistent actions by powerful professional bodies, including the Health Care Accreditation Council (HCAC), the Jordanian Nurses and Midwives Association (JNMA), and the Jordan Nursing Council (JNC), in implementing policies that focus on professional growth, lifelong learning, and career progression. Such programs aim at creating a highly competent nursing staff that can function at its best in advanced and complex healthcare environments [23].

These findings are consistent with Alanazi et al. [11] in Saudi Arabia, where a descriptive study using Kelley’s followership model among 355 nurses found that 74% were exemplary followers and 19% were pragmatists. The same trends were observed in a cross-cultural study with participants in Canada, where authors found that 59.5% of the sample were exemplary followers, 35.1% were pragmatists, and 2.7% were conformist or passive followers, with no alienated followers found [12]. Moreover, Boothe et al. [13] in the United States reported that 93% of nurses were exemplary, 5% were pragmatists, and only a small proportion were conformists (2%), with no alienated or passive followers identified. In Korea, by contrast, indicated considerably disparate distributions: 18% exemplary, 36% pragmatists, 8% alienated, 17% conformists, 21% passive [24]. These variations strengthen the assertion that followership styles and perceptions differ significantly across cultural groups [8–10]^.^

Although exemplary followership was the most prominent style in this current study, most respondents were unaware of their followership style. This finding shows a mismatch between behavior demonstration and self-awareness, and the majority of nurses exercising effective followership without overt conceptual knowledge of the follower role [25]. This may be an underlying reason due to a lack of a widely accepted definition of nursing followership that describes role expectations and distinguishes between effective and ineffective followership nursing behaviors [25,26]. Without this definitional clarity, nurses may be exhibiting effective followership yet still be unable to label or define their style.

This is in line with the broader literature highlighting that followership remains under-theorized and underemphasized throughout healthcare education and practice [1,11,26,27]. Besides, nurses prefer to conceptualize their professional identity in either a clinical practice frame of reference or a leadership orientation, rather than being leader followership strategic contributors [1,2,27]. Therefore, followership as a professional construct can tend to be either invisible or disregarded, even for highly-performing nursing professionals.

Building on the overall distribution of followership styles identified in this study, further analysis was conducted to explore how these styles varied according to demographic and workplace-related characteristics among nurses in Jordan. The present study found considerable difference between followership styles and demographic- workplace characteristics, including marital status, unit type, nursing care delivery model, and decision-making style.

The findings revealed that single and separated nurses were more likely to demonstrate exemplary followership than married nurses. These results imply that marital status may shape the way nurses engage in followership behaviors, possibly due to varying levels of autonomy, flexibility, commitment, or external responsibilities [10,28]. The greater prevalence of exemplary followership among single or separated nurses may be explained by their greater autonomy and fewer family related responsibilities, allowing them to invest more cognitive and emotional efforts into their professional roles. On the other hand, based on the Jordanian culture that values family-oriented life, married nurses may experience interfering responsibilities related to family life, which can moderate their levels of engagement at work and influence their alignment with more pragmatic followership styles characterized by moderate engagement and independence critical thinking. This association aligns with broader literature on work-life balance and professional role strain [28].

This evidence-based research finding appropriately aligns with the recent study conducted by Dalky et al. [29], whose findings explained that, among single nurses, there was a higher amount of engagement in error reporting practices as a result of their less complicated work-life balance and enhanced autonomy, which improve their proactive professional behaviors as one of the main indicators of exemplary followership. On the same note, the same patterns were found in other countries. Alqarawi et al. [30] discovered that nurses who are younger, female and unmarried in Saudi Arabia were more likely to see toxic leadership, which showed that these demographic categories were not only more responsive to their working conditions but also potentially more proactive in their jobs as they did not have many complicated personal issues. Although the Jordanian culture values family-oriented life, this result indicates that active engagement is possible due to individual autonomy, which is one of the most noticeable features of single nurses. Although this interpretation is exploratory, no previous studies, to our knowledge, have directly examined the association between marital status and followership style, highlighting the need for further targeted research

Furthermore, the working area emerged as a significant contextual factor influencing followership. Nurses in emergency departments demonstrated a markedly higher likelihood of exhibiting exemplary followership compared with those in ICUs or general wards. This pattern aligns with the highly dynamic, fast-paced, and autonomous Emergency Department’s work setting, which often demands timely decision-making capacity, adaptability, and proactive engagement with the multidisciplinary team, all hallmarks of exemplary followership [9,30]. Conversely, ICU environments, while also complex, tend to be more protocol-driven and hierarchical, which may limit opportunities for autonomous behavior [12]. This consistent with previous study Zhang et al. [31] who found low level on nurse engagement among ICU nurses due to high pressure and stressful work environment, while a recent scoping review similarly identified reduced autonomy among ICU nurses working within physician-dominated collaborative models, often linked to unclear or restricted professional role boundaries [31,32]. To our knowledge, no previous studies have directly compared followership styles across ED and ICU settings, which underscores the novelty of this finding and highlights its contribution to the emerging empirical literature on nursing followership.

In addition, the current study found that the nurses who working in team-based care and functional units exhibited exemplary followership, and those working under unclear care models embraced a more pragmatic style. These results are explained in light of existing literature, as clear expectations regarding roles and responsibilities are crucial in maintaining the required competence, knowledge, skills, and caring behaviour [26]. When nurses understand their roles within the team and how they contribute to the overall goals of the organization, they are more likely to engage actively and take ownership of their responsibilities [33–35]. Providing clarity on performance expectations fosters a sense of accountability, motivating nurses to demonstrate effective followership behaviours [36]. In addition, A culture that support teamwork and shared goals, fosters a sense of belonging and commitment among nurses, enhancing their willingness to engage actively in their roles.

In the scope of the global perspective, Owens et al. [37] also pointed out a well-organized environment and well-formed expectations contributing to autonomous decision-making process which allows them to practice within their full scope of practice, the latter of which is a precondition of effective exemplary followership.

Nationally, these findings align with Smama’h et al. [38], who established that supportive and achievement-oriented leadership styles characterized by role clarity correlate positively with motivation, which could consequently lead to exemplary followership, literature supported the positive relation between motivation and increased level of work engagement [39]. A lack of well-designed and clearly defined care usually creates ambiguity, leading to the reduction of motivation or clarity needed to be followed proactively.

Also, this present study found a significant difference between decision-making style and followership behaviors. Its findings show that nurses who worked in the participatory system or mixed environment of decision-making were much more likely to be outstanding followers. These findings may be attributed to the fact that nurses who feel valued and involved in the decision-making process are high likely to engage in effective followership behaviors. Conversely, authoritarian leadership styles that exclude nurses from decisions, make unilateral decisions, and enforce strict control over their tasks can inhibit such engagement [40]. This result is congruent with the findings of a Canadian study by Peabody et al. [12], which reported a shift in healthcare followership from a traditional vertical hierarchy where followers were viewed as passive subordinates toward a more horizontal structure characterized by active follower engagement in participatory decision-making processes.

Moreover, the significant difference identified between effective followership and participatory decision-making can be understood in light of leadership behaviors. Nationally, the finding supported the results of the study by Alkarabsheh et al. [41], which stresses the importance of transformational and authentic leadership in promoting professional engagement by supporting follower participation by inviting feedback, respecting different opinions, and involving staff nurses in decision-making processes. The same finding was achieved by Zhang et al. [31] in China, whereby transformational leadership, which is inclusive and empowering, highly contributes to work engagement, which is a major characteristic of outstanding followers. These leadership behaviors create a supportive environment that encourages followers to think critically and contribute actively. This may explain why nurses who are included in decisions are more likely to show effective followership behaviors.

Nonetheless, no significant associations were found between followership style and demographic variables such as age, gender, income, or educational level, nor with workplace characteristics such as shift type or hospital sector. These findings partially differ from those of Alanazi et al. [11], who reported that higher education was positively associated with exemplary followership and that male nurses were more likely to adopt passive or pragmatic styles. Such discrepancies may reflect contextual and cultural differences between healthcare systems. In Jordan, nursing practice is embedded within relatively homogeneous organizational structures characterized by standardized role expectations and hierarchical leadership practices [15,16], which may attenuate the influence of individual demographic traits on behavioral tendencies. Moreover, contemporary conceptualizations of followership emphasize its dynamic nature and its sensitivity to factors such as role clarity, team relationships, and situational leadership demands [1,26]. From this perspective, the absence of demographic predictors in the present study aligns with the view that followership behaviors are shaped more by workplace context and relational dynamics than by fixed demographic characteristics [42].

In the present study, neither most types of healthcare sector nor geographical region emerged as significant independent predictors of exemplary followership. This result suggests that macro-structural factors exert less influence on nurses’ followership behaviors than unit-level relational and organizational conditions. Contemporary evidence indicates that effective followership is shaped primarily by proximal workplace dynamics—such as leadership style, psychological safety, role clarity, and autonomy—rather than by hospital ownership or geographic location [1,26,28,32]. Studies from Jordan similarly show that nurses’ professional outcomes, including engagement, satisfaction, and performance, are more strongly determined by the quality of the immediate work environment than by sectoral or regional differences [4,5,16,38]. Moreover, all major healthcare sectors in Jordan operate under unified national regulations, accreditation requirements, and licensing standards set by the Ministry of Health and the Jordan Nursing Council. Across these sectors, nurses face similar workforce shortages, heavy workloads, and hierarchical organizational structures, resulting in largely comparable professional challenges regardless of geographic region. Under these shared conditions, sector or location is less likely to create meaningful differences in opportunities for independent critical thinking or active engagement. Instead, unit-level factors—such as leadership style, decision-making processes, and local organizational culture—play a more influential role in shaping exemplary followership, many of which were already captured within the regression model. These findings support the interpretation that exemplary followership is not inherently sector- or region-dependent but emerges from supportive, autonomy-enhancing, and healthy work environment cultures across all healthcare settings.

### Strength and limitations

This study offers several notable strengths that contribute meaningfully to both academic literature and practical applications. A major strength of this study lies in its rigorous and methodologically robust multistage sampling strategy, which ensured balanced representation across Jordan’s geographic regions, healthcare sectors, and hospital types, thereby enhancing external validity. By including nurses from 15 hospitals in the northern, middle, and southern regions, encompassing governmental, private, and university-affiliated institutions, the study achieved a high level of national representation, strengthening the generalizability of its findings. Data collection was efficient and accessible, utilizing a Google Forms online survey distributed through professional WhatsApp channels, enabling timely engagement with frontline nurses across diverse hospital settings. Valid and reliable instruments were used to assess the three primary variables under investigation.

This study is subject to several limitations. First, all data were obtained through self-administered questionnaires, which are inherently vulnerable to common method biases such as social desirability and recall bias. Consequently, self-reported data may not accurately reflect the actual characteristics of the workplace. Additionally, although nursing units were randomly selected, recruitment relied on voluntary participation from nurses present during data-collection periods, which may introduce self-selection bias. While practical, it may limit the representativeness of the sample.

### Implications and recommendations

Nursing development agencies should explicitly include followership as a core professional competency, recognizing its value in fostering accountability, shared responsibility, and improved patient outcomes. An organizational culture that supports effective followership can enhance job performance, staff engagement, and professional satisfaction. In Jordan, there is an urgent need for continuous professional development programs to raise awareness among healthcare providers about their followership styles and to strengthen their leadership capacity in practice.

Based on the findings of this study, further research is strongly recommended to deepen the understanding of followership styles within nursing and multidisciplinary team contexts. Qualitative methodologies, such as descriptive phenomenological studies, in-depth interviews, focus groups, and unstructured interviews, could be particularly valuable for uncovering the underlying barriers and facilitators to effective followership in healthcare organizations. Although the KFQ-R cut-points used in this study follow Kelley’s original recommendations [17], future research in Middle Eastern contexts should empirically evaluate whether these thresholds adequately capture the full range of followership behaviors across diverse cultural and organizational environments.

Incorporating structured followership education into nursing curricula is essential for preparing graduates to function as both effective team members and future leaders. In the Jordanian context, a targeted educational initiative could include integrating followership competencies into existing leadership and communication courses, using case-based learning and high-fidelity simulation to practice active engagement, assertive communication, and decision-making within team scenarios. Additionally, guided reflective activities and debriefing sessions can help students examine follower–leader dynamics, clarify role expectations, and develop the critical thinking and situational judgment required for effective followership in real clinical environments. Collaboration between policymakers, regulatory bodies, and nurse educators is critical for developing leadership competencies rooted in participative principles. Managers can foster participatory decision-making by establishing shared governance councils, conducting regular interdisciplinary huddles, and creating unit-based committees that invite nurses’ input on workflow and quality issues. When nurses are invited to voice their ideas, see their input reflected in decisions, and receive clear updates about unit performance, they feel genuinely valued and become more confident in contributing to the team’s direction.

## Conclusion

This study revealed that most nurses exhibited behaviors aligned with the exemplary followership style, characterized by active engagement and independent critical thinking. Nevertheless, a considerable proportion were unaware of their own followership style, indicating a gap between behavioral practice and self-perception. The findings also highlighted significant associations between followership styles and contextual factors such as the model of care delivery, decision-making structures, and work setting. Nurses working in participatory environments, team-based models of care, and high-intensity units (e.g., emergency departments) were more likely to demonstrate exemplary followership behaviors. These settings appear to foster autonomy, critical thinking, and active involvement in team processes, thereby reinforcing effective followership. Strengthening nurses’ awareness of followership concepts through targeted educational and leadership development initiatives, alongside supportive organizational structures, may further enhance collaboration, professional growth, and the overall quality of healthcare delivery.

## Supporting information

S1 File Survey instrument used in this study. (PDF)

S2 File STROBE checklist. (PDF)
