# Supplementary material for: Followership among jordanian nurses: A cross-sectional online study
Source: PLoS One. 2025 Dec 23;20(12):e0339008. doi: 10.1371/journal.pone.0339008 (PMC12725662; doi:10.1371/journal.pone.0339008)
Supplement: S1 File — (PDF) [file pone.0339008.s001.pdf]

KFQ-R tool

Identifying Your Followership Style

Since most of us spend the majority of our time in the followership role, it stands to reason that how we perform as followers determines, for the most part, how satisfied we are with our day-to-day work existence

This questionnaire will help you determine your style of followership, as well as identify your strengths and areas for development as a follower. This is a self-diagnostic instrument, so please respond to each statement as you naturally behave.

For each statement, please use the scale below to indicate the extent to which the statement describes you. Think of a specific but typical followership situation and how you acted.

0	1	2	3	4	5	6
Rarely			Occasionally			Almost Always

_____1. I think about how my work adds to society

_____2. I spend time thinking about how my work contributes to my personal fulfillment?

_____3. Alignment between my personal and organizational goals helps me stay involved at work?

_____4. Does your enthusiasm also spread to and energize your peers?

_____5. I contribute my best at work

_____6. Do you actively develop a distinctive competence in those critical activities so that you become more valuable to the organization and its leaders?

_____7. I evaluate activities that are necessary for organizational goal achievement?

_____8. I develop competencies in my work to increase my value to the organization?

_____9. Do you take the initiative to seek out and successfully complete assignments that go above and beyond your role?

_____10. The leader can give me an assignment without supervision, knowing that I will complete it?

0	1	2	3	4	5	6
Rarely			Occasionally			Almost Always

_____11. I finish assignments that go beyond my job duties?

_____12. Do you try to solve the tough problems (technical, organizational, etc) rather than look to the leader to do it for you?

_____13. I generate and evaluate new ideas that contribute to the organizational goals?

_____14. I try to solve problems rather than rely on the leader?

_____15. Do you understand the leader’s needs, goals, and constraints, and work hard to meet them?

_____16. Do you actively and honestly own up to your strengths and weaknesses rather than put off evaluation?

_____17. I help my team to see the potential and risks of ideas and plans?

_____18. When the leader asks you to do something that runs contrary to your preferences, do you say “no” rather than “yes?”

_____19. I work to achieve the leader's needs and goals?

_____20. Do you assert your views in important issues, even though it might mean conflict with your group or leader?

_____21. I question internally the wisdom of the leader’s decisions?

_____22. I do what the leader requests regardless of my beliefs?

_____23. I act on my own ethical standards rather than those of my work group (team)?

_____24. I assert my views on important issues, even though they may conflict with coworkers?

_____25. Do you assert your views in important issues, even though it might mean conflict with your group or leader?
