# Supplementary material for: Followership among jordanian nurses: A cross-sectional online study
Source: PLoS One. 2025 Dec 23;20(12):e0339008. doi: 10.1371/journal.pone.0339008 (PMC12725662; doi:10.1371/journal.pone.0339008)
Supplement: S2 File — (PDF) [file pone.0339008.s002.pdf]

STROBE Statement—Checklist of items that should be included in reports of ***cross-sectional studies***

	Item No	Recommendation	Location in Manuscript (Page/Section)
**Title and abstract**	1	(*a*) Indicate the study's design with a commonly used term in the title or the abstract	Page 1<br>Line 2
		(*b*) Provide in the abstract an informative and balanced summary of what was done and what was found	Page 2<br>Line 20-48
**Introduction**			
Background/rationale	2	Explain the scientific background and rationale for the investigation being reported	Page 3 – 6<br>Line 49- 131<br>background paragraph page (1-3)<br>Gap in Paragraph page(4-5)<br>Significance in paragraph page (5)
Objectives	3	State specific aims and objectives	Page 6<br>Line 117-131<br>Aim in paragraph page (6) line 117
**Methods**			
Study design	4	Present key elements of study design early in the paper	Page 7<br>Line 133-143 in first paragraph under method heading.
Setting	5	Describe the setting, locations, and relevant dates, including periods of recruitment, exposure, follow-up,<br><br>and data collection	Page 7<br>Line 133-143<br><br>Page 10<br>Line 210-220
Participants	6	(*a*) Give the eligibility criteria, and the sources and methods of selection of participants	Page 7<br>144-156
Variables	7	Clearly define all outcomes, exposures, predictors, potential confounders, and effect modifiers. Give diagnostic criteria, if applicable	Page 8 - 9<br>Line 167-191
Data sources/ measurement	8*	For each variable of interest, give sources of data and details of methods of assessment (measurement). Describe comparability of assessment methods if there is more than one group	*Page 8 - 9*<br>*Line 167-191*
Bias	9	Describe any efforts to address potential sources of bias	Page 7<br>Line 134 -156
Study size	10	Explain how the study size was arrived at	Page 8<br>Line 157-166
Quantitative variables	11	Explain how quantitative variables were handled in the analyses. If applicable, describe which groupings	Page 8 - 9<br>Line 167-191

		were chosen and why	
Statistical methods	12	(*a*) Describe all statistical methods, including those used to control for confounding	Page 11<br>Line 232- 244
		(*b*) Describe any methods used to examine subgroups and interactions	Page 11<br>Line 232- 244
		(*c*) Explain how missing data were addressed	Page 11<br>Line 232- 244
		(*d*) If applicable, describe analytical methods taking account of sampling strategy	
**Results**			
Participants	13*	(a) Report numbers of individuals at each stage of study—eg numbers potentially eligible, examined for eligibility, confirmed eligible, included in the study, completing follow-up, and analysed	Page 11-21<br>Line 245-380
		(b) Give reasons for non-participation at each stage	N/A
		(c) Consider use of a flow diagram	N/A
Descriptive data	14*	(a) Give characteristics of study participants (eg demographic, clinical, social) and information on exposures and potential confounders	Page 12-13<br>Line 246-277
		(b) Indicate number of participants with missing data for each variable of interest	Page 12-13<br>Line 246-277
Outcome data	15*	Report numbers of outcome events or summary measures	Page 11-21<br>Line 245-380
Main results	16	(*a*) Give unadjusted estimates and, if applicable, confounder-adjusted estimates and their precision (eg, 95% confidence interval). Make clear which confounders were adjusted for and why they were included	Page 11-21<br>Line 245-380
		(*b*) Report category boundaries when continuous variables were categorized	Page 11-21<br>Line 245-380
		(*c*) If relevant, consider translating estimates of relative risk into absolute risk for a meaningful time period	Not relevant
Other analyses	17	Report other analyses done—eg analyses of subgroups and interactions, and sensitivity analyses	
**Discussion**			
Key results	18	Summarise key results with reference	Page 22-29

		to study objectives	Line 382 - 562
Limitations	19	Discuss limitations of the study, taking into account sources of potential bias or imprecision. Discuss both direction and magnitude of any potential bias	Page 30<br>Line 578-584
Interpretation	20	Give a cautious overall interpretation of results considering objectives, limitations, multiplicity of analyses, results from similar studies, and other relevant evidence	
Generalisability	21	Discuss the generalisability (external validity) of the study results	Page 30<br>Line 565 - 577
**Other information**			
Funding	22	Give the source of funding and the role of the funders for the present study and, if applicable, for the original study on which the present article is based	Page 33<br>Line 635 -636

*Give information separately for exposed and unexposed groups.
